# Survival Outcomes and Patterns of Care for Stage II or III Resected Gastric Cancer by Race and Ethnicity

**DOI:** 10.1001/jamanetworkopen.2023.49026

**Published:** 2023-12-21

**Authors:** S. Peter Wu, Sara H. Keshavjee, Sam S. Yoon, Steve Kwon

**Affiliations:** 1 Department of Radiation Oncology, City of Hope Medical Center, Duarte, California; 2 Department of Surgery, McMaster University, Hamilton, Ontario, Canada; 3 Department of Surgery, Columbia University Medical Center, New York, New York; 4 Department of Surgery, Roger Williams Medical Center and Boston University, Providence, Rhode Island

## Abstract

**Importance:**

Many multimodality treatment regimens exist for gastric adenocarcinoma, including neoadjuvant vs adjuvant chemotherapy, radiation, or both. Neoadjuvant therapy is recommended in the United States for patients with locally advanced gastric cancer; however, it is unknown whether the outcomes of neoadjuvant therapy are associated with race and ethnicity.

**Objective:**

To evaluate the differences in outcomes by race and ethnicity of patients with noncardia gastric cancer undergoing surgical procedures with and without neoadjuvant therapy.

**Design, Setting, and Participants:**

This retrospective cohort study examined the National Cancer Database from the American College of Surgeons for patients with clinical stage II or III gastric adenocarcinoma, excluding gastric cardia tumors, undergoing surgical resection procedures from January 2006 to December 2019. Statistical analysis was performed from December 2021 to May 2023.

**Exposure:**

Patients were stratified by race and ethnicity, and their outcomes were analyzed for those who received and did not receive neoadjuvant therapy.

**Main Outcomes and Measures:**

The Cox proportional hazard model was used to compare overall survival (OS) between racial and ethnic groups (Asian, Black, Hispanic, and White) overall and according to receipt of neoadjuvant therapy. Among those who received neoadjuvant therapy, proportional differences in pathological responses were calculated in each group.

**Results:**

Among a total of 6938 patients in the cohort, 4266 (61.4%) were male; mean (SD) age was 65.9 (12.8) years; 1046 (15.8%) were Asian, 1606 (24.3%) were Black, 1175 (17.8%) were Hispanic, and 3540 (53.6%) were White. Compared with other races and ethnicities, the group of White patients had significantly more who were 65 years or older with more comorbidities. White patients underwent surgical resection procedures alone without neoadjuvant or adjuvant therapy more frequently than other races and ethnicities. Asian and Black patients had the highest proportion of being downstaged or achieving pathological complete response after neoadjuvant therapy. In multivariate models, perioperative chemotherapy was associated with improved OS (HR, 0.79 [95% CI, 0.69-0.90]), whereas number of positive lymph nodes and surgical margins were associated with the largest decreases in OS. Asian and Hispanic race and ethnicity were associated with significantly improved OS compared with Black and White races (eg, Asian patients: HR, 0.64 [95% CI, 0.58-0.72]; and Hispanic patients: HR, 0.77 [95% CI, 0.69-0.85]). Black race was associated with improved OS compared with White race when receiving neoadjuvant therapy (HR, 0.78 [95% CI, 0.67-0.90]).

**Conclusions and Relevance:**

In this large nationwide cohort study of survival outcomes among patients with resected clinical stage II or III gastric cancer, there were significant differences in response to treatment and OS between different racial and ethnic groups.

## Key Points

**Question:**

Among patients with stage II or III gastric cancer who underwent a curative surgical procedure, are there outcome differences by race and ethnicity?

**Findings:**

In this cohort study of 6938 patients with clinical stage II or III noncardia gastric adenocarcinoma, Asian and Hispanic patients had statistically significantly better overall survival (OS) compared with White patients. Black patients were observed to have similar OS with White patients overall, but Black patients did have statistically significantly better OS among those who received neoadjuvant therapy.

**Meaning:**

Among patients with stage II or III gastric adenocarcinoma undergoing a curative surgical procedure, there were variations in OS associated with race and ethnicity.

## Teaser

This cohort study evaluates the differences in outcomes by race and ethnicity of patients with noncardia gastric cancer undergoing surgical procedures with and without neoadjuvant therapy.

## Introduction

Gastric cancer is the fifth most common cancer in the world with more than 26 500 new diagnoses and 11 130 deaths annually in the United States.^1^ Although the incidence has declined, some studies suggest that there is increasing incidence and mortality in younger patients,^2^ and 5-year survival rate is still poor at 30%.^3^ Surgical resection is potentially curative; however, variation in extent of lymph node dissection and type of perioperative chemotherapy and radiation therapy have led to numerous paradigms for therapeutic sequencing in advanced resectable gastric cancer.

The updated analysis of the Intergroup 0116 trial demonstrated a strong persistent overall survival benefit from adjuvant chemoradiation compared with surgery alone (hazard ratio [HR], 1.32 [95% CI, 1.10-1.60]).^4^ However, most patients who entered in this trial underwent less than a D2 lymphadenectomy, and many have argued that the chemoradiation compensated for inadequate node dissection. This argument was strengthened with the ARTIST 2 trial^5^ indicating no benefit of adding radiotherapy to adjuvant chemotherapy after D2 lymphadenectomy in patients with stage II or III node-positive gastric cancer. Perioperative chemotherapy became an established treatment regimen based on the MAGIC trial,^6^ which showed a significant improvement in 5-year overall and progression-free survival compared with surgery alone. This was followed by the FLOT4 trial,^7^ which established perioperative FLOT (fluorouracil plus leucovorin, oxaliplatin, and docetaxel) as the new preoperative standard chemotherapy over MAGIC’s ECF (fluorouracil or capecitabine plus cisplatin and epirubicin) regimen. These studies have changed our practice pattern for locally advanced gastric cancer over the past decade but variabilities in use of neoadjuvant or adjuvant treatment protocols have existed across the United States due to different rates of adoption of these studies.

Studies have also found significant disparities in gastric cancer outcomes across racial and ethnic groups in the United States. One study hypothesized that a substantial driver of disparity is the fact that Asian, Black, Hawaiian or Pacific Islander, and Hispanic patients are more likely than non-Hispanic White patients to develop noncardia, diffuse-type gastric cancer, which is associated with treatment resistance and worse outcomes.^8^ There are data to suggest population differences in molecular subtypes of gastric cancer according to race, affecting therapeutic response and prognosis.^9^ Minoritized racial and ethnic populations may also face systems-level differences in care leading to worse outcomes.^10,11^ Others have questioned whether response to neoadjuvant or adjuvant chemotherapy differs across racial and ethnic groups.^12,13^ Our current study was designed to review the recent patterns of care in resectable gastric cancer in the United States, compared with survival outcomes of patients by race and ethnicity, and analyze differences in outcomes after different multimodality treatment regimens stratified by race and ethnicity.

## Methods

### Data

This was a retrospective cohort study using the National Cancer Database (NCDB), which is a hospital-based registry of cancer outcomes produced by the American Cancer Society and the American College of Surgeon’s Commission on Cancer (CoC). The deidentified database includes data collected from teaching hospitals, community cancer centers, and other cancer centers including Veterans Affairs hospitals located in 49 states and Puerto Rico. Only CoC-approved hospitals are allowed to report data to the NCDB and an estimated 70% of all new diagnoses of cancer in the United States are captured by the database. The NCDB Participant User Data File (PUF) is a Health Insurance Portability and Accountability Act–compliant data file containing cases submitted to the CoC’s NCDB and waives informed consent process as the PUF only contains deidentified patient level data. This analysis used data available to CoC-accredited facilities that lacked personal identifiers and were exempt from institution review board approval owing to use of publicly available, deidentified data, in accordance with 45 CFR §46. We followed the Strengthening the Reporting of Observational Studies in Epidemiology (STROBE) reporting guideline.

### Cohort

The NCDB for gastric cancer contained 255 165 patients. The NCDB was queried for patients treated in 2006 to 2019 with American Joint Committee on Cancer clinical stage IIA to IIIC gastric adenocarcinoma who underwent partial or total gastrectomy, excluding patients with cancers of the gastric cardia due to overlap with Siewert II and III malignant neoplasms of the gastroesophageal junction. For the purpose of this study, we excluded those with previous history of cancer, those who did not undergo gastrectomy, and those who had pathology different from adenocarcinoma. We also excluded those without information on sequencing of radiation, chemotherapy, and surgical resection procedure. Lastly, because we were interested in association with neoadjuvant or adjuvant therapy, we excluded those with stage 0 to I and stage IV gastric cancers as well as those with unknown clinical stage. This left us with 7545 patients, of which 6938 patients had complete follow-up with survival data.

### Variables

Variables available for analyses included age, sex, race and ethnicity, Charlson Deyo score, primary site, histology, grade, stage, extent of lymph node dissection, number of lymph nodes positive, treatment facility type, distance from treatment facility to zip code, insurance status, treatment regimen and sequence, type of surgical procedure, and residual tumor resection classification. Patient’s race and ethnicity were determined from predefined NCDB data based on assignment by a CoC registrar according to fixed categories. For the race and ethnicity variable, we created 4 large categories: Asian, Black, Hispanic, and White. We grouped the Asian race category to include East Asian, South Asian, and Pacific Islander. A small portion of those who did not fall into the 4 categories of race and ethnicity were grouped as having other race and ethnicity. The multimodality treatment modality variable was categorized as neoadjuvant chemotherapy only, perioperative chemotherapy, neoadjuvant chemoradiation only, perioperative chemotherapy with radiation (either adjuvant or neoadjuvant), adjuvant chemotherapy only, adjuvant chemoradiation only, and surgical procedure only. In comparing neoadjuvant vs no neoadjuvant chemotherapy groups, we grouped the neoadjuvant chemotherapy group to contain any form of chemotherapy prior to surgical procedure (ie, neoadjuvant chemotherapy only, perioperative chemotherapy, neoadjuvant chemoradiation, perioperative chemotherapy with radiation). Among those who received neoadjuvant therapy, we compared the clinical stage and the final pathological stage, and then categorized response to neoadjuvant therapy.

### Statistical Analysis

Univariate Cox regression analyses were performed to assess for variables that were associated with overall survival (OS). Variables with *P* < .05 on the univariate model and had more than 20 events per variable were included in the multivariate model. Median follow-up was estimated by the method of Schemper and Smith.^14^ All analyses were conducted from December 2021 to May 2023 using R version 4.1.2 (R Project for Statistical Computing). All tests were 2-sided with *P* < .05 as the threshold for significance.

## Results

Among a total of 6938 patients in the cohort, 4266 (61.4%) were male; mean (SD) age was 65.9 (12.8) years; 1046 (15.8%) were Asian, 1606 (24.3%) were Black (24.3%); 1175 (17.8%) Hispanic; 3540 (53.6%) were White (53.6%), 178 (2.6%) were other race or ethnicity, and 333 (4.8%) were missing race and ethnicity information (Table 1). There was a higher proportion of patients aged at least 65 years among White patients (66.0% [2338 of 3540]) compared with patients from other racial and ethnic groups, with Hispanic patients having the lowest proportion (44.6% [524 of 1175]). Black and White patients had significantly more comorbidities compared with Asian and Hispanic patients (Table 1). Higher proportions of Asian patients received treatment in academic tertiary centers (51.7% [541 of 1046]) with facilities being less than 10 miles from their home address (68.5% [717 of 1046]) compared with other racial and ethnic groups (eTable 1 in Supplement 1). In terms of insurance status, lower proportions of White patients were uninsured (1.6% [56 of 3540]) and had Medicaid (4.9% [172 of 3540]) compared with Hispanic patients (uninsured: 11.2% [132 of 1175]; Medicaid: 17.7% [208 of 1175]) (Table 1).

**Table 1.** Sociodemographic and Clinical Variables by Race and Ethnicity for Clinical Stage II or III Gastric Cancer

Variables	Patients, No. (%)					*P* value
	Asian	Black	Hispanic	White	Other^a^	
No.	1046	1606	1175	3540	178	NA
Age, y						
<50	111 (10.6)	184 (11.5)	248 (21.1)	275 (7.8)	17 (9.6)	<.001
50-65	355 (33.9)	644 (40.1)	403 (34.3)	927 (26.2)	61 (34.3)	
≥65	580 (55.4)	778 (48.4)	524 (44.6)	2338 (66.0)	100 (56.2)	
Sex						
Male	676 (64.6)	1008 (62.8)	737 (62.7)	2129 (60.1)	121 (68.0)	.02
Female	370 (35.4)	598 (37.2)	438 (37.3)	1411 (39.9)	57 (32.0)	
Comorbidity index						
0	758 (72.5)	1057 (65.8)	852 (72.5)	2397 (67.7)	129 (72.5)	<.001
1	214 (20.5)	383 (23.8)	231 (19.7)	782 (22.1)	42 (23.6)	
2	44 (4.2)	101 (6.3)	54 (4.6)	238 (6.7)	5 (2.8)	
≥3	30 (2.9)	65 (4.0)	38 (3.2)	123 (3.5)	2 (1.1)	
Insurance status						
Private	360 (34.4)	552 (34.4)	422 (35.9)	1138 (32.1)	63 (35.4)	<.001
Medicare	451 (43.1)	713 (44.4)	383 (32.6)	2092 (59.1)	75 (42.1)	
Medicaid	165 (15.8)	200 (12.5)	208 (17.7)	172 (4.9)	25 (14.0)	
Uninsured	49 (4.7)	91 (5.7)	132 (11.2)	56 (1.6)	7 (3.9)	
Other government	4 (0.4)	24 (1.5)	7 (0.6)	35 (1.0)	4 (2.2)	
Unknown	17 (1.6)	26 (1.6)	23 (2.0)	47 (1.3)	4 (2.2)	
Primary site						
Fundus	46 (4.4)	49 (3.1)	62 (5.3)	256 (7.2)	7 (3.9)	<.001
Body	110 (10.5)	203 (12.6)	152 (12.9)	475 (13.4)	31 (17.4)	
Antrum	402 (38.4)	563 (35.1)	366 (31.1)	965 (27.3)	55 (30.9)	
Pylorus	39 (3.7)	80 (5.0)	49 (4.2)	168 (4.7)	3 (1.7)	
Lesser curvature	175 (16.7)	258 (16.1)	184 (15.7)	493 (13.9)	35 (19.7)	
Greater curvature	40 (3.8)	66 (4.1)	74 (6.3)	220 (6.2)	10 (5.6)	
Overlapping lesion	122 (11.7)	168 (10.5)	142 (12.1)	451 (12.7)	18 (10.1)	
Unknown	112 (10.7)	219 (13.6)	146 (12.4)	512 (14.5)	19 (10.7)	
Surgical margins						
Negative (R0)	899 (85.9)	1388 (86.4)	969 (82.5)	2916 (82.4)	158 (88.8)	<.001
Positive microscopic (R1)	75 (7.2)	87 (5.4)	92 (7.8)	288 (8.1)	13 (7.3)	
Positive macroscopic (R2)	5 (0.5)	5 (0.3)	12 (1.0)	26 (0.7)	1 (0.6)	
Positive NOS	39 (3.7)	99 (6.2)	77 (6.6)	217 (6.1)	5 (2.8)	
Unknown	28 (2.7)	27 (1.7)	25 (2.1)	93 (2.6)	1 (0.6)	
Regional nodes examined						
No. of nodes, mean (SD)	24.5 (15.0)	20.6 (13.0)	22.4 (14.2)	19.7 (12.9)	21.9 (14.4)	<.001
Clinical stage						
II	77 (7.4)	146 (9.1)	103 (8.8)	405 (11.4)	13 (7.3)	<.001
IIA	299 (28.5)	463 (28.8)	312 (26.6)	970 (27.4)	52 (29.2)	
IIB	261 (24.9)	390 (24.3)	313 (26.7)	896 (25.4)	56 (31.4)	
III	131 (12.5)	135 (8.4)	122 (10.4)	220 (6.2)	15 (8.5)	
IIIA	154 (14.7)	277 (17.2)	183 (15.6)	633 (17.9)	23 (12.9)	
IIIB	87 (8.3)	142 (8.8)	99 (8.4)	288 (8.1)	11 (6.2)	
IIIC	37 (3.5)	53 (3.3)	43 (3.7)	128 (3.6)	8 (4.5)	
Treatment						
Neoadjuvant chemotherapy	245 (23.4)	412 (25.7)	290 (24.7)	819 (23.1)	50 (28.1)	<.001
Adjuvant chemotherapy	143 (13.7)	196 (12.2)	142 (12.1)	341 (9.6)	19 (10.7)	
Adjuvant chemoradiation	199 (19.0)	327 (20.4)	229 (19.5)	606 (17.1)	35 (19.7)	
Perioperative chemotherapy	176 (16.8)	205 (12.8)	185 (15.7)	483 (13.6)	30 (16.9)	
Surgical resection only	213 (20.4)	345 (21.5)	222 (18.9)	981 (27.7)	33 (18.5)	
Neoadjuvant chemotherapy plus radiation	30 (2.9)	54 (3.4)	40 (3.4)	174 (4.9)	6 (3.4)	
Perioperative chemotherapy with radiation (neoadjuvant or adjuvant)	40 (3.8)	67 (4.2)	67 (5.7)	136 (3.8)	5 (2.8)	

Abbreviations: NA, not applicable; NOS, not otherwise specified.

^a^ The National Cancer Database lists races and ethnicities as other if they cannot be categorized as American Indian/Aleutian/Eskimo, Asian Indian, Black, Chamorran, Chinese, Fiji Islander, Filipino, Japanese, Guamanian, Hawaiian, Hmong, Kampuchean, Korean, Laotian, Melanesian, Micronesian, New Guinean, Other Asian, Pacific Islander, Pakistani, Polynesian, Samoan, Spanish/Hispanic, Tahitian, Thai, Tongan, Vietnamese, or White.

There were significantly higher proportions of signet cell or diffuse type histology in the Hispanic group (16.8% [197 of 1175]) (eTable 1 in Supplement 1), whereas White patients had significantly higher proportion of cancers in the fundus (7.2% [256 of 3540]) (Table 1). Correspondingly, White (82.4% [2916 of 3540]) and Hispanic (82.5% [969 of 1175]) patients had the lowest R0 surgical margin resection rates. The mean (SD) lymph nodes examined were highest in the Asian patient group (24.5 [15.0]) with 42.7% (447 of 1046) having had more than 25 lymph nodes examined, and lowest in the White patient group (19.7 [12.9]) with 29.7% (1052 of 3540) having had more than 25 lymph nodes examined) (Table 1; eTable 1 in Supplement 1). White patients had the highest proportion who underwent surgical resection alone (27.7% [981 of 3540]), with Hispanic patients having the lowest proportion (18.9% [222 of 1175]). Asian patients had the highest proportion that received perioperative chemotherapy (16.8% [176 of 1046]), with Black patients having the lowest proportion (12.8% [205 of 1606]) (Table 1). It should also be noted that even though certain minor differences between different racial and ethnic groups achieved statistical significance in the current sample size, they may not reflect clinical relevance.

When looking at trends over time, the use of perioperative chemotherapy increased over time (2.5% [6 of 242] in 2006 to 30.7% [186 of 605] by 2019) as well as neoadjuvant chemotherapy (8.3% [20 of 242] in 2006 to 39.0% [236 of 605] by 2019). In contrast, surgical resection alone was used less frequently (34.7% [84 of 242] in 2006 to 8.6% [52 of 605] by 2019) as well as adjuvant chemoradiation (34.7% [84 of 242] in 2006 to 4.1% [25 of 605] by 2019). There were also increasing numbers of regional lymph nodes being examined, and rates of more than 25 lymph nodes examined went from 19.8% [48 of 242] in 2006 to 45.5% [275 of 605] in 2019 (Table 2).

**Table 2.** Sociodemographic and Clinical Variables Over Time Among Patients With Clinical Stage II or III Gastric Cancer

Variables	Patients, No. (%)														*P* value
	2006	2007	2008	2009	2010	2011	2012	2013	2014	2015	2016	2017	2018	2019	
No.	242	261	405	426	581	633	634	666	669	581	600	621	621	605	NA
Age, y															
<50	33 (13.6)	31 (11.9)	33 (8.1)	49 (11.5)	61 (10.5)	74 (11.7)	89 (14.0)	66 (9.9)	73 (10.9)	53 (9.1)	60 (10.0)	64 (10.3)	72 (11.6)	77 (12.7)	.006
50-65	71 (29.3)	76 (29.1)	109 (26.9)	125 (29.3)	162 (27.9)	199 (31.4)	194 (30.6)	204 (30.6)	209 (31.2)	200 (34.4)	217 (36.2)	197 (31.7)	205 (33.0)	222 (36.7)	
≥65	138 (57.0)	154 (59.0)	263 (64.9)	252 (59.2)	358 (61.6)	360 (56.9)	351 (55.4)	396 (59.5)	387 (57.8)	328 (56.5)	323 (53.8)	360 (58.0)	344 (55.4)	306 (50.6)	
Sex															
Male	146 (60.3)	144 (55.2)	239 (59.0)	275 (64.6)	362 (62.3)	381 (60.2)	368 (58.0)	413 (62.0)	414 (61.9)	371 (63.9)	367 (61.2)	383 (61.7)	405 (65.2)	403 (66.6)	.05
Race and ethnicity															
Asian	33 (13.6)	34 (13.0)	36 (8.9)	53 (12.4)	64 (11.0)	75 (11.8)	68 (10.7)	89 (13.4)	88 (13.2)	64 (11.0)	99 (16.5)	107 (17.2)	109 (17.6)	127 (21.0)	<.001
Black	46 (19.0)	57 (21.8)	86 (21.2)	91 (21.4)	131 (22.5)	112 (17.7)	144 (22.7)	145 (21.8)	142 (21.2)	117 (20.1)	134 (22.3)	138 (22.2)	124 (20.0)	139 (23.0)	
Hispanic	41 (16.9)	32 (12.3)	45 (11.1)	57 (13.4)	77 (13.3)	92 (14.5)	108 (17.0)	111 (16.7)	93 (13.9)	85 (14.6)	107 (17.8)	108 (17.4)	111 (17.9)	108 (17.9)	
White	120 (49.6)	138 (52.9)	227 (56.0)	217 (50.9)	299 (51.5)	342 (54.0)	307 (48.4)	309 (46.4)	318 (47.5)	301 (51.8)	240 (40.0)	245 (39.5)	259 (41.7)	218 (36.0)	
Other^a^	2 (0.8)	0 (0.0)	11 (2.7)	8 (1.9)	10 (1.7)	12 (1.9)	7 (1.1)	12 (1.8)	28 (4.2)	14 (2.4)	20 (3.3)	23 (3.7)	18 (2.9)	13 (2.1)	
Lymph nodes examined															
0	5 (2.1)	20 (7.7)	14 (3.5)	24 (5.6)	36 (6.2)	17 (2.7)	18 (2.8)	13 (2.0)	18 (2.7)	16 (2.8)	8 (1.3)	8 (1.3)	15 (2.4)	9 (1.5)	<.001
1-14	115 (47.5)	94 (36.0)	178 (44.0)	177 (41.5)	226 (38.9)	243 (38.4)	219 (34.5)	211 (31.7)	212 (31.7)	164 (28.2)	139 (23.2)	130 (20.9)	111 (17.9)	95 (15.7)	
15-25	74 (30.6)	87 (33.3)	114 (28.1)	121 (28.4)	181 (31.2)	215 (34.0)	215 (33.9)	212 (31.8)	220 (32.9)	196 (33.7)	207 (34.5)	221 (35.6)	206 (33.2)	226 (37.4)	
>25	48 (19.8)	60 (23.0)	99 (24.4)	104 (24.4)	138 (23.8)	158 (25.0)	182 (28.7)	230 (34.5)	219 (32.7)	205 (35.3)	246 (41.0)	262 (42.2)	289 (46.5)	275 (45.5)	
Treatment															
Neoadjuvant chemotherapy	20 (8.3)	24 (9.2)	43 (10.6)	55 (12.9)	69 (11.9)	113 (17.9)	99 (15.6)	155 (23.3)	176 (26.3)	166 (28.6)	214 (35.7)	217 (34.9)	229 (36.9)	236 (39.0)	<.001
Adjuvant chemotherapy	32 (13.2)	35 (13.4)	40 (9.9)	48 (11.3)	63 (10.8)	75 (11.8)	83 (13.1)	83 (12.5)	91 (13.6)	54 (9.3)	60 (10.0)	64 (10.3)	64 (10.3)	49 (8.1)	
Adjuvant chemoradiation	84 (34.7)	84 (32.2)	127 (31.4)	114 (26.8)	150 (25.8)	172 (27.2)	163 (25.7)	139 (20.9)	109 (16.3)	87 (15.0)	61 (10.2)	54 (8.7)	27 (4.3)	25 (4.1)	
Perioperative chemotherapy	6 (2.5)	5 (1.9)	12 (3.0)	19 (4.5)	40 (6.9)	58 (9.2)	56 (8.8)	73 (11.0)	99 (14.8)	75 (12.9)	130 (21.7)	138 (22.2)	182 (29.3)	186 (30.7)	
Surgical resection only	84 (34.7)	94 (36.0)	160 (39.5)	165 (38.7)	222 (38.2)	173 (27.3)	181 (28.5)	169 (25.4)	149 (22.3)	127 (21.9)	72 (12.0)	86 (13.8)	60 (9.7)	52 (8.6)	
Neoadjuvant chemoradiation	7 (2.9)	14 (5.4)	15 (3.7)	10 (2.3)	15 (2.6)	24 (3.8)	31 (4.9)	23 (3.5)	19 (2.8)	37 (6.4)	28 (4.7)	29 (4.7)	28 (4.5)	24 (4.0)	
Perioperative chemotherapy with radiation	9 (3.7)	5 (1.9)	8 (2.0)	15 (3.5)	22 (3.8)	18 (2.8)	21 (3.3)	24 (3.6)	26 (3.9)	35 (6.0)	35 (5.8)	33 (5.3)	31 (5.0)	33 (5.5)	

Abbreviation: NA, not applicable.

^a^ The National Cancer Database lists races and ethnicities as other if they cannot be categorized as American Indian/Aleutian/Eskimo, Asian Indian, Black, Chamorran, Chinese, Fiji Islander, Filipino, Japanese, Guamanian, Hawaiian, Hmong, Kampuchean, Korean, Laotian, Melanesian, Micronesian, New Guinean, Other Asian, Pacific Islander, Pakistani, Polynesian, Samoan, Spanish/Hispanic, Tahitian, Thai, Tongan, Vietnamese, or White.

**Figure 1.**
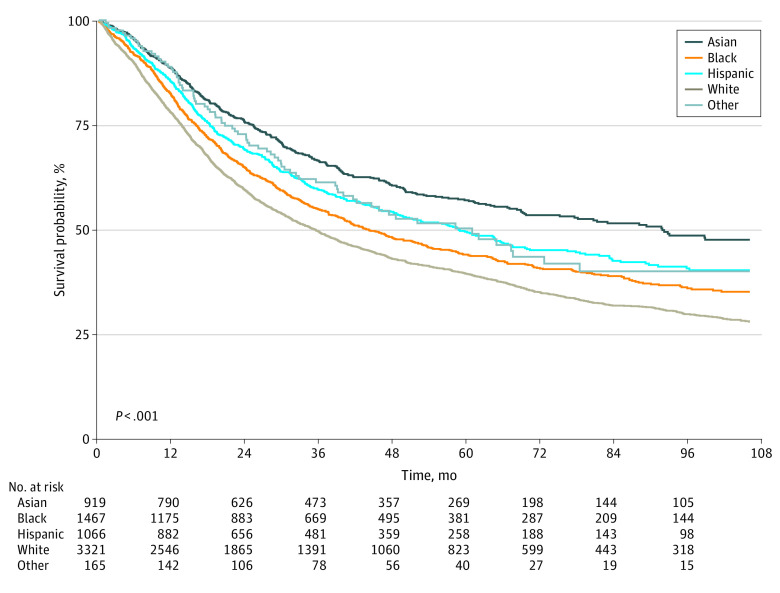
Overall Survival of Clinical Stage II or III Gastric Adenocarcinoma Patients by Race

When looking at unadjusted OS of patients with clinical stage II or III cancer by race and ethnicity, we found that Asian and Hispanic patients had significantly better survival than White patients (*P* < .001 for both Asian and Hispanic patients) (Figure 1). Furthermore, when comparing OS of these patients by receipt of some form of neoadjuvant chemotherapy in their treatment regimen sequence, we found that those who received neoadjuvant chemotherapy treatment did significantly better than those who did not receive neoadjuvant chemotherapy (eFigure in Supplement 1). In the multivariate analyses, perioperative chemotherapy was associated with improved OS (HR, 0.79 [95% CI, 0.69-0.90]) (Table 3). In addition, after adjusting for sociodemographic and clinical covariates, including type of multimodality treatment regimen, race and ethnicity was a significant factor in OS. The adjusted OS was significantly higher in Asian patients with 36% lower risk of death compared with White patients (HR, 0.64 [95% CI, 0.58-0.72]) (Table 3). The OS for Hispanic patients as compared with White patients also persisted with multivariate adjustment (HR, 0.77 [95% CI, 0.69-0.85]). Other variables associated with differences in OS included age, comorbidities, facility type, insurance status, histology, lymphovascular invasion, surgical margins, clinical stage, number of lymph nodes positive, response to neoadjuvant therapy, and type of multimodality treatment (Table 3).

**Table 3.** Multivariate Analysis of Hazard Ratios for Sociodemographic and Clinical Variables

Variables	Hazard ratio (95% CI)		
	All patients	Patients receiving neoadjuvant therapy	Patients not receiving neoadjuvant therapy
Age, y			
<50	1 [Reference]	1 [Reference]	1 [Reference]
50-65	1.14 (1.00-1.31)	1.11 (0.90-1.37)	1.14 (0.95-1.37)
≥65	1.25 (1.08-1.45)	1.19 (0.93-1.51)	1.31 (1.08-1.59)
Sex			
Female	1 [Reference]	1 [Reference]	1 [Reference]
Male	1.00 (0.93-1.06)	0.98 (0.87-1.10)	0.98 (0.91-1.06)
Comorbidity index			
0	1 [Reference]	1 [Reference]	1 [Reference]
1	1.07 (0.99-1.15)	1.01 (0.88-1.16)	1.07 (0.97-1.18)
2	1.20 (1.06-1.37)	1.02 (0.79-1.32)	1.25 (1.07-1.45)
≥3	1.44 (1.21-1.71)	1.65 (1.19-2.29)	1.38 (1.11-1.70)
Insurance status			
Private	1 [Reference]	1 [Reference]	1 [Reference]
Medicare	1.17 (1.07-1.29)	0.99 (0.84-1.17)	1.27 (1.12-1.43)
Medicaid	0.98 (0.86-1.11)	0.89 (0.72-1.09)	1.08 (0.92-1.27)
Uninsured	0.96 (0.80-1.15)	1.31 (1.00-1.73)	0.90 (0.71-1.14)
Other government	1.33 (0.97-1.82)	1.51 (0.95-2.41)	1.23 (0.80-1.89)
Unknown	0.85 (0.65-1.12)	1.04 (0.65-1.67)	0.80 (0.57-1.12)
Facility type			
Academic	1 [Reference]	1 [Reference]	1 [Reference]
Community	1.22 (1.06-1.40)	1.03 (0.77-1.40)	1.27 (1.08-1.48)
Comprehensive community cancer program	1.16 (1.07-1.25)	1.07 (0.93-1.22)	1.20 (1.10-1.32)
Integrated network	1.14 (1.04-1.26)	1.10 (0.94-1.30)	1.16 (1.03-1.30)
Unknown	1.10 (0.88-1.39)	1.04 (0.75-1.46)	1.06 (0.76-1.47)
Surgical margins			
Negative	1 [Reference]	1 [Reference]	1 [Reference]
Positive macroscopic	1.98 (1.44-2.72)	3.16 (1.48-6.76)	2.08 (1.46-2.95)
Positive microscopic	1.74 (1.57-1.93)	1.97 (1.64-2.37)	1.66 (1.46-1.89)
Positive NOS	1.65 (1.47-1.86)	1.75 (1.40-2.20)	1.69 (1.47-1.95)
Unknown	1.31 (1.07-1.60)	1.21 (0.84-1.74)	1.37 (1.07-1.75)
Clinical stage			
II	1 [Reference]	1 [Reference]	1 [Reference]
III	1.40 (1.29-1.52)	1.31 (1.13-1.52)	1.58 (1.14-2.20)
Lymph nodes positive			
0 Lymph nodes	1 [Reference]	1 [Reference]	1 [Reference]
1-3 Lymph nodes	1.35 (1.22-1.49)	1.52 (1.28-1.79)	1.41 (1.25-1.60)
4-9 Lymph nodes	1.77 (1.58-1.99)	1.83 (1.49-2.25)	2.12 (1.86-2.42)
≥10 Lymph nodes	2.95 (2.59-3.36)	3.27 (2.62-4.09)	3.56 (3.06-4.14)
Unknown	1.61 (1.12-2.31)	1.72 (0.89-3.32)	1.81 (1.17-2.80)
Multimodality treatment			
Neoadjuvant chemotherapy	1 [Reference]	1 [Reference]	NA
Adjuvant chemotherapy	0.96 (0.85-1.08)	NA	1 [Reference]
Adjuvant chemoradiation	0.83 (0.74-0.93)	NA	0.84 (0.75-0.94)
Perioperative chemotherapy	0.79 (0.69-0.90)	0.73 (0.64-0.84)	NA
Surgical resection only	1.79 (1.62-1.97)	NA	1.66 (1.48-1.86)
Neoadjuvant chemoradiation	1.26 (1.06-1.50)	1.30 (1.09-1.56)	NA
Perioperative chemoradiation	0.98 (0.82-1.16)	0.95 (0.79-1.13)	NA
Response to neoadjuvant treatment			
No response	1 [Reference]	1 [Reference]	NA
Pathological complete response	0.60 (0.33-1.09)	0.59 (0.31-1.12)	NA
Downstaged	0.64 (0.57-0.73)	0.72 (0.60-0.86)	NA
Upstaged	1.30 (1.17-1.45)	1.24 (1.04-1.48)	NA
Metastatic	1.80 (1.58-2.04)	1.77 (1.42-2.21)	NA
Unknown	0.83 (0.75-0.93)	0.74 (0.61-0.89)	NA
Race and ethnicity			
Asian	0.64 (0.58-0.72)	0.55 (0.45-0.68)	0.68 (0.59-0.78)
Black	0.96 (0.88-1.04)	0.78 (0.67-0.90)	1.06 (0.95-1.17)
Hispanic	0.77 (0.69-0.85)	0.74 (0.63-0.88)	0.76 (0.67-0.87)
White	1 [Reference]	1 [Reference]	1 [Reference]
Other^a^	0.82 (0.66-1.03)	0.87 (0.62-1.22)	0.74 (0.54-1.00)

Abbreviations: NA, not applicable; NOS, not otherwise specified.

^a^ The National Cancer Database lists races and ethnicities as other if they cannot be categorized as American Indian/Aleutian/Eskimo, Asian Indian, Black, Chamorran, Chinese, Fiji Islander, Filipino, Japanese, Guamanian, Hawaiian, Hmong, Kampuchean, Korean, Laotian, Melanesian, Micronesian, New Guinean, Other Asian, Pacific Islander, Pakistani, Polynesian, Samoan, Spanish/Hispanic, Tahitian, Thai, Tongan, Vietnamese, or White.

**Figure 2.**
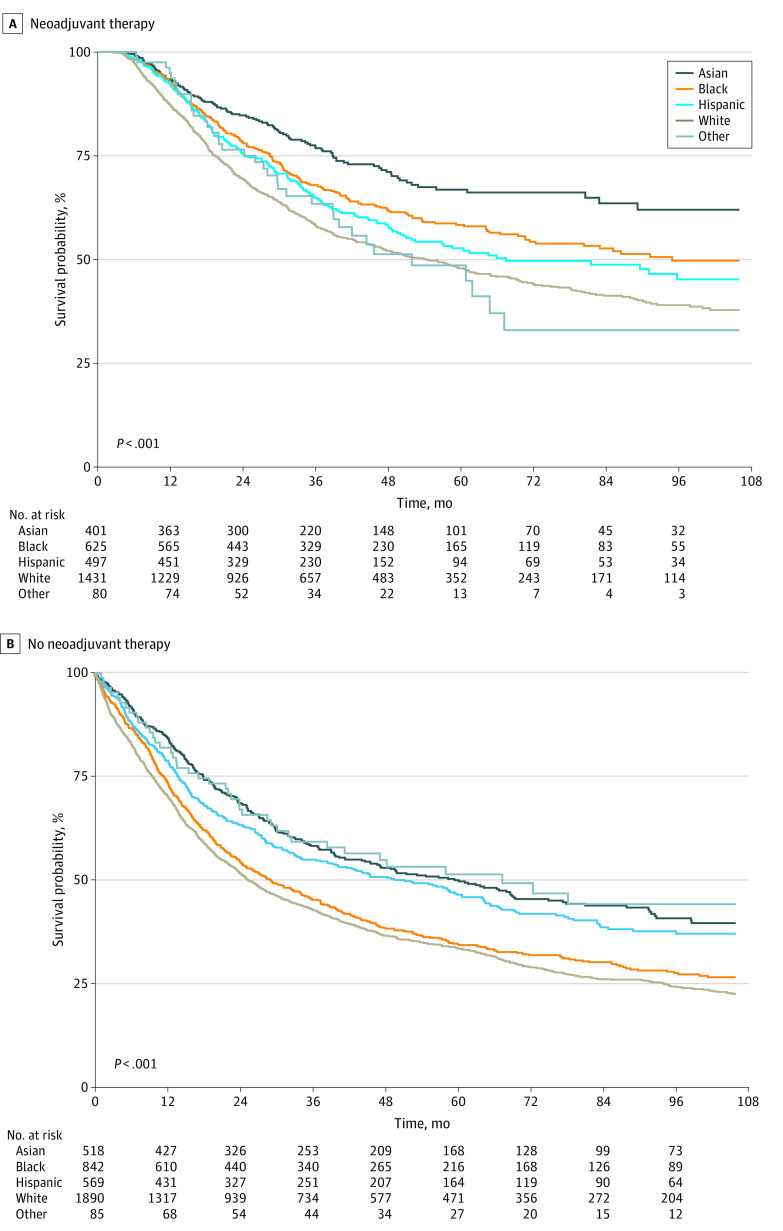
Overall Survival of Clinical Stage II or III Gastric Adenocarcinoma With and Without Neoadjuvant Therapy by Race

To explore whether there was also an association of race and ethnicity and neoadjuvant therapy with OS, we compared the OS of our cohort who received some form of neoadjuvant chemotherapy by race and ethnicity (Figure 2A) and those who did not receive any form of neoadjuvant chemotherapy by race and ethnicity (Figure 2B). Black patients who received neoadjuvant chemotherapy had OS that appeared similar to Hispanic patients (Figure 2A), whereas Black patients who did not receive neoadjuvant chemotherapy had OS falling to be similar to that of White patients (Figure 2B). Similarly, although Black patients had a similar HR with White patients when looking at the whole cohort (HR, 0.96 [95% CI, 0.88-1.04]), Black race was associated with improved OS when only looking at the group of patients who received neoadjuvant therapy (HR, 0.78 [95% CI, 0.67-0.90]) (Table 3). Interestingly, Black patients had the highest proportion of being downstaged or achieving pathological complete response after neoadjuvant chemotherapy (34.4%) along with Asian patients (35.3%) when compared with White (28.4%) and Hispanic (30.8%) patients. Meanwhile, Hispanic patients had the highest proportion of being upstaged (19.1%) or becoming metastatic (5.4%) after neoadjuvant therapy (eTable 2 in Supplement 1).

## Discussion

In this study of 6938 patients in the NCDB with stage II or III gastric adenocarcinoma undergoing surgical resections, there are wide variations and statistically significant changes in the use of neoadjuvant and adjuvant therapy over time, and differential outcomes in treatment response and OS associated with race and ethnicity. Among all multimodality regimens, perioperative chemotherapy was associated with improved OS, whereas the number of positive lymph nodes and positive surgical margin were associated with the lowest OS. Among all patients in our cohort, Asian and Hispanic patients had improved OS compared with Black and White patients. Among those who received neoadjuvant therapy, Black patients were associated with higher OS compared with White patients. Asian and Black patients had higher proportions responding favorably to neoadjuvant therapy, with downstaging or pathological complete response, compared with White patients.

This retrospective cohort study of the NCDB found that race and ethnicity were independently associated with gastric cancer outcomes, even when adjusted for sociodemographic and clinical factors. Specifically, Asian and Hispanic patients had improved survival outcomes compared with Black and White patients. Other studies have reported similar results; an earlier NCDB study of 47 217 patients between 2000 to 2015 found that patients who self-identified as Asian had improved survival outcomes, followed by Black patients, then White patients.^15^ Various factors may drive this difference, such as differences in tumor response to chemotherapy or lymph node metastasis rate. Multiple studies have shown that Asian patients have a significantly reduced frequency of lymph node metastases, which is a significant factor for survival as demonstrated in our multivariate analysis when compared with White or Black patients.^15,16,17^ Our study, however, did not find that Asian and Hispanic patients had higher lymph node metastasis compared with Black and White patients with clinical stage II or III gastric cancer. Some studies have also shown that Asian patients were more likely to receive adequate lymphadenectomy.^18^ We also found that Asian and Hispanic patients had a higher number of regional lymph nodes examined. However, this may not be the sole driver of improved outcomes across different racial and ethnic groups. A study using the SEER database of more than 12 000 patients found that the survival benefit persisted after adjustment for age, gender, tumor grade, and number of examined and positive lymph nodes.^19^ Similarly, a study of more than 47 000 patients from the California Cancer Registry found that Asian and Hispanic race and ethnicity were strong independent factors of survival.^20^ Our study also found that race and ethnicity is an important independent factor of survival in multivariate analysis, with Asian patients having the lowest HR, aligned with the results of prior studies.

This study found that better survival outcomes were associated with patients with stage II or III gastric adenocarcinoma treated with neoadjuvant therapy, particularly perioperative chemotherapy, which has been found in a meta-analysis of randomized clinical trials.^21^ Fortunately, we are seeing an increasing proportion of patients receiving some form of neoadjuvant therapy over time and declining use of postoperative chemoradiotherapy, indicating appropriate adoption of treatment standards established by clinical trials over time. However, disparities in receipt of multimodality therapy in the United States have been shown. Using the NCDB, Al-Refaie et al^10^ found that between 1998 and 2006, when use of neoadjuvant chemotherapy for gastric adenocarcinoma was not standard practice, Black and Hispanic patients were less likely to receive multimodal therapy with adjuvant chemotherapy compared with White patients.^10^ More recently, a study of more than 16 000 patients using NCDB from 2006 to 2014 found that non-Hispanic White patients were more likely to receive neoadjuvant treatment than Asian or Pacific Islander, Black, or Hispanic patients.^11^ They found that race and ethnicity other than non-Hispanic White was also associated with no insurance and low education level, which were both independently associated with lower rates of receiving neoadjuvant treatment.^11^ In the present study, although lack of insurance or Medicaid insurance was more common in Asian, Black, and Hispanic patient populations compared with White patients, we did not find disparities in receipt of multimodality treatment with White patients having the highest proportion of patients who underwent surgical resection only without multimodality treatment. This may have been due to White patients presenting at older age as we observed significantly higher proportions of those at least 65 years of age. Correspondingly, White patients also had the worst OS outcomes.

As it is still unclear what is most strongly contributing to race and ethnicity–based differences in survival outcomes, some have speculated that individuals may differ in degree of response to chemotherapy. Various genetic sequence variants have been correlated with increased or decreased response to neoadjuvant chemotherapy for gastric cancer,^13^ and it is possible that these sequence variants have different frequencies across racial and ethnic groups. A small prospective study the from University of California San Diego found that Asian race was independently associated with increased likelihood to respond to neoadjuvant therapy and achieve a pathologic complete response when compared with Black or White patients.^12^ The authors suggested possible reasons such as variations in molecular subtype frequencies with differences in chemotherapy response and a difference in host immune response.^12^ In our study, the use of neoadjuvant treatment was found to be associated with better OS, and with some similarity to results from Rajabnejad et al,^12^ the rate of downstaging or complete pathological response was significantly higher in the Asian and Black patient groups as compared with the White patient group. Interestingly, although Black patients overall had similar OS compared with White patients, Black patients had higher OS compared with White patients only among those who received neoadjuvant therapy.

### Limitations

While this study has advantages in its large sample size with diverse patient populations across the United States, it also has limitations. The NCDB only collects data from CoC-accredited facilities, which may capture approximately 70% of cancer diagnoses. Therefore, its generalizability may be limited. This study reports race and ethnicity–based outcomes. There may be misclassification of certain racial and ethnic groups, and certain patients may belong to 2 or more different racial and ethnic groups which are unaccounted for in this study. Furthermore, it is possible that certain racial disparities that arise from differences in access to CoC-accredited hospitals are unaccounted for. Additionally, due to data limitation, we were only able to compare clinical stage to pathological stage obtained postoperatively to compare response to neoadjuvant therapy. Furthermore, clinical stage in NCDB is captured through review of physician’s record and/or coding information in the patient record. This may have introduced misclassification of staging groups; however, we found no evidence to suggest that there were differential misclassifications of clinical stages or pathological stages of certain racial or ethnic groups.

## Conclusions

To our knowledge, this is the first large nationwide retrospective cohort study evaluating the association of neoadjuvant therapy with different racial and ethnic groups with clinical stage II and III gastric cancer. Asian and Black patient groups had higher proportions of patients responding favorably to neoadjuvant therapy with downstaging or pathological complete response, which may contribute to Black patients having higher OS compared with White patients among those who received neoadjuvant therapy. Interestingly, Asian and Hispanic race and ethnicity were independently associated with improved OS compared with Black and White race, even after adjustment including multimodality treatment regimen and responses to neoadjuvant therapy. Our findings suggest the importance of future research toward investigating the biological differences in tumor behavior across races that have yet to be elucidated, including consideration of clinical trial design in gastric cancer as well as genomic studies to include stratification on factors of race and ethnicity.

## Notes

Supplement 1. **eTable 1.** Additional Sociodemographic and Clinical Variables by Race/Ethnicity **eTable 2.** Comparison of Initial Clinical Staging to Pathologic Staging of Patients After Receiving Neoadjuvant Therapy Followed by Surgery **eFigure.** Survival Probability With Neoadjuvant Therapy Vs Without

Supplement 2.
Data Sharing Statement
