# Supplementary material for: Survival Outcomes and Patterns of Care for Stage II or III Resected Gastric Cancer by Race and Ethnicity
Source: JAMA Netw Open. 2023 Dec 21;6(12):e2349026. doi: 10.1001/jamanetworkopen.2023.49026 (PMC10739152; doi:10.1001/jamanetworkopen.2023.49026)
Supplement: Supplement 1. — eTable 1. Additional Sociodemographic and Clinical Variables by Race/Ethnicity eTable 2. Comparison of Initial Clinical Staging to Pathologic Staging of Patients After Receiving Neoadjuvant Therapy Followed by Surgery eFigure. Survival Probability With Neoadjuvant Therapy Vs Without [file jamanetwopen-e2349026-s001.pdf]

Supplemental Online Content

Wu SP, Keshavjee SH, Yoon SS, Kwon S. Survival outcomes and patterns of care for stage II or III resected gastric cancer by race and ethnicity. *JAMA Netw Open.* 2023;6(12):e2349026. doi:10.1001/jamanetworkopen.2023.49026

eTable 1. Additional Sociodemographic and Clinical Variables by Race/Ethnicity

eTable 2. Comparison of Initial Clinical Staging to Pathologic Staging of Patients After Receiving Neoadjuvant Therapy Followed by Surgery

eFigure. Survival Probability With Neoadjuvant Therapy vs Without

This supplemental material has been provided by the authors to give readers additional information about their work.

© 2023 Wu SP et al. *JAMA Network Open.*

eTable 1. Additional sociodemographic and clinical variables by race/ethnicity

	Asian	Black	Hispanic	White	Other	P value
**N**	1046	1606	1175	3540	178	
**Facility Type, No. (%)**						<0.001
Academic	541 (51.7)	717 (44.6)	580 (49.4)	1475 (41.7)	100 (56.2)	
Community	57 (5.4)	80 (5.0)	56 (4.8)	205 (5.8)	4 (2.2)	
Comprehensive Community Cancer Program	290 (27.7)	499 (31.1)	304 (25.9)	1187 (33.5)	33 (18.5)	
Integrated Network	126 (12.0)	253 (15.8)	149 (12.7)	615 (17.4)	37 (20.8)	
**Distance From Facility in miles, No. (%)**						<0.001
<10	717 (68.5)	1057 (65.8)	761 (64.8)	1788 (50.5)	95 (53.4)	
10-20	175 (16.7)	245 (15.3)	198 (16.9)	681 (19.2)	39 (21.9)	
20-50	96 (9.2)	184 (11.5)	125 (10.6)	631 (17.8)	22 (12.4)	
50-100	32 (3.1)	74 (4.6)	38 (3.2)	251 (7.1)	8 (4.5)	
>100	26 (2.5)	46 (2.9)	53 (4.5)	189 (5.3)	14 (7.9)	
**Histology, No. (%)**						<0.001
Signet Cell or Diffuse	115 (11.0)	115 (7.2)	197 (16.8)	339 (9.6)	17 (9.6)	
Adenocarcinoma Intestinal	257 (24.6)	344 (21.4)	232 (19.7)	624 (17.6)	40 (22.5)	
Adenocarcinoma, NOS	674 (64.4)	1147 (71.4)	746 (63.5)	2577 (72.8)	121 (68.0)	
**Lymphovascular invasion, No. (%)**						0.06
Absent	327 (31.3)	541 (33.7)	392 (33.4)	1121 (31.7)	60 (33.7)	
Present	375 (35.9)	539 (33.6)	449 (38.2)	1247 (35.2)	70 (39.3)	
Unknown/indeterminate	344 (32.9)	526 (32.8)	334 (28.4)	1172 (33.1)	48 (27.0)	
**Grade, No. (%)**						0.007
Well Differentiated	32 (3.1)	60 (3.7)	29 (2.5)	103 (2.9)	5 (2.8)	
Moderately Differentiated	280 (26.8)	497 (30.9)	282 (24.0)	944 (26.7)	50 (28.1)	
Poorly Differentiated	687 (65.7)	962 (59.9)	800 (68.1)	2317 (65.5)	112 (62.9)	
Unknown	47 (4.5)	87 (5.4)	64 (5.4)	176 (5.0)	11 (6.2)	
**Surgery Type, No. (%)**						<0.001
Gastrectomy, NOS	264 (25.3)	468 (29.2)	302 (25.7)	858 (24.3)	48 (27.0)	
Antrectomy (<40% of stomach)	44 (4.2)	50 (3.1)	29 (2.5)	108 (3.1)	2 (1.1)	
Distal Gastrectomy	337 (32.2)	416 (25.9)	260 (22.1)	748 (21.1)	47 (26.4)	
Proximal Gastrectomy	24 (2.3)	59 (3.7)	35 (3.0)	119 (3.4)	7 (3.9)	
Near-total or total gastrectomy, NOS	14 (1.3)	21 (1.3)	13 (1.1)	41 (1.2)	1 (0.6)	
Near-total Gastrectomy	19 (1.8)	23 (1.4)	20 (1.7)	72 (2.0)	1 (0.6)	
Total Gastrectomy	147 (14.1)	223 (13.9)	223 (19.0)	623 (17.6)	30 (16.9)	
Gastrectomy, NOS w/portion of esophagus	8 (0.8)	19 (1.2)	11 (0.9)	76 (2.1)	3 (1.7)	
Partial Gastrectomy w/portion of esophagus	48 (4.6)	84 (5.2)	69 (5.9)	219 (6.2)	10 (5.6)	
Total Gastrectomy w/portion of esophagus	29 (2.8)	37 (2.3)	39 (3.3)	149 (4.2)	5 (2.8)	
Gastrectomy NOS w/other organs	15 (1.4)	18 (1.1)	22 (1.9)	83 (2.3)	3 (1.7)	
Partial Gastrectomy w/other organs	55 (5.3)	113 (7.0)	69 (5.9)	190 (5.4)	12 (6.7)	
Total Gastrectomy w/other organs	25 (2.4)	54 (3.4)	53 (4.5)	175 (4.9)	5 (2.8)	
Radical Gastrectomy w/other organs	17 (1.6)	21 (1.3)	30 (2.6)	79 (2.2)	4 (2.2)	
**Regional Nodes Examined, No. (%)**						<0.001
0 Lymph Nodes	21 (2.0)	45 (2.8)	27 (2.3)	127 (3.6)	1 (0.6)	
1-14 Lymph Nodes	235 (22.5)	501 (31.2)	337 (28.7)	1179 (33.3)	62 (34.8)	
15-25 Lymph Nodes	343 (32.8)	560 (34.9)	357 (30.4)	1182 (33.4)	53 (29.8)	
>25 Lymph Nodes	447 (42.7)	500 (31.1)	454 (38.6)	1052 (29.7)	62 (34.8)	
**Regional Nodes Positive, No. (%)**						<0.001
0 positive node	333 (31.8)	524 (32.6)	358 (30.5)	1208 (34.1)	55 (30.9)	
1-3 positive nodes	276 (26.4)	464 (28.9)	298 (25.4)	932 (26.3)	58 (32.6)	
4-9 positive nodes	218 (20.8)	345 (21.5)	254 (21.6)	730 (20.6)	40 (22.5)	
≥10 positive nodes	190 (18.2)	216 (13.4)	223 (19.0)	511 (14.4)	19 (10.7)	
Unknown	29 (2.8)	57 (3.5)	42 (3.6)	159 (4.5)	6 (3.4)	

© 2023 Wu SP et al. *JAMA Network Open.*

eTable 2. Comparison of initial clinical staging to pathologic staging of patients after receiving neoadjuvant therapy followed by surgery

Variables	ypCR	Downstaged	Same Stage	Upstaged	Metastatic	Unknown
Asian, No. (%)	9 (2.2)	104 (25.9)	125 (31.2)	67 (16.7)	15 (3.7)	81 (20.2)
Black, No. (%)	10 (1.6)	169 (27.0)	221 (35.4)	98 (15.7)	23 (3.7)	104 (16.6)
Hispanic, No. (%)	4 (0.8)	124 (24.9)	165 (33.2)	95 (19.1)	27 (5.4)	82 (16.5)
White, No. (%)	19 (1.3)	322 (22.5)	543 (37.9)	251 (17.5)	64 (4.5)	232 (16.2)
Other, No. (%)	1 (1.3)	23 (28.8)	30 (37.5)	16 (20)	1 (1.3)	9 (11.3)

Variables	ypCR or Downstaged	Same Stage/Upstaged/ Metastatic
Asian, No. (%)	113 (33.4)	225 (66.6)
Black, No. (%)	179 (32.6)	370 (67.4)
Hispanic, No. (%)	128 (29.4)	308 (70.6)
White, No. (%)	341 (27.0)	920 (73.0)
Other, No. (%)	24 (31.6)	52 (68.4)

© 2023 Wu SP et al. *JAMA Network Open.*

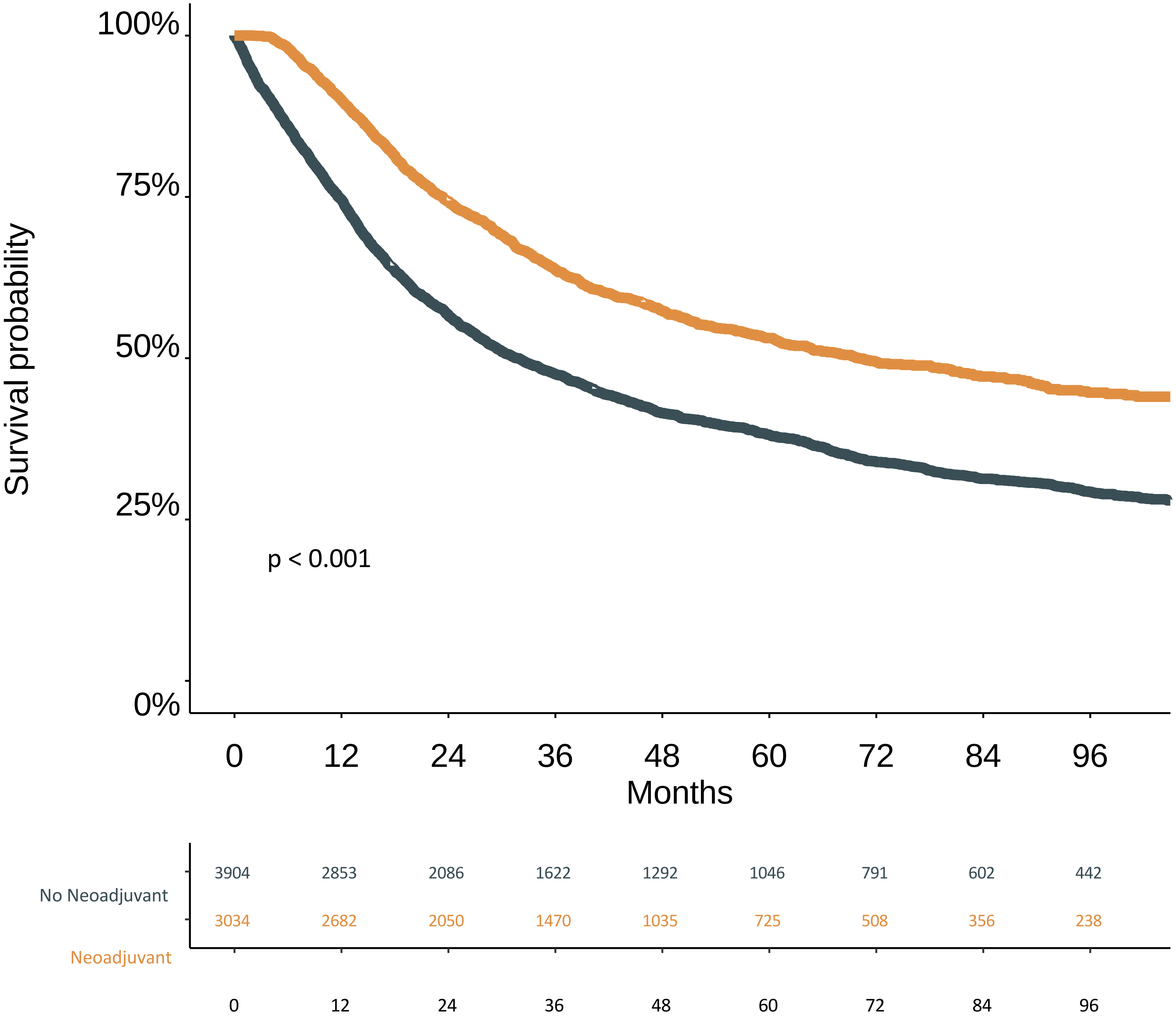

© 2023 Wu SP et al. *JAMA Network Open.*

Months

No Neoadjuvant Neoadjuvant

eFigure. Survival Probability With Neoadjuvant Therapy vs Without

© 2023 Wu SP et al. *JAMA Network Open.*
