# Supplementary material for: Survival Outcomes and Patterns of Care for Stage II or III Resected Gastric Cancer by Race and Ethnicity
Source: JAMA Netw Open. 2023 Dec 21;6(12):e2349026. doi: 10.1001/jamanetworkopen.2023.49026 (PMC10739152; doi:10.1001/jamanetworkopen.2023.49026)
Supplement: Supplement 2. — Data Sharing Statement [file jamanetwopen-e2349026-s002.pdf]

Data Sharing Statement

Wu. Survival Outcomes and Patterns of Care for Stage II or III Resected Gastric Cancer by Race and Ethnicity. *JAMA Netw Open*. Published December 21, 2023.
doi:10.1001/jamanetworkopen.2023.49026

Data
Data available: Yes
Data types: Deidentified participant data
How to access data: https://www.facs.org/quality-programs/cancer-programs/national-cancer-database/puf/
When available: With publication

Supporting Documents
Document types: None

Additional Information
Who can access the data: investigators associated with CoC-accredited cancer programs.
Types of analyses: For any purpose
Mechanisms of data availability: With signed data access agreement with American College of Surgeons
